# Improving the Extraction of Polyphenols from Cocoa Bean Shells by Ultrasound and Microwaves: A Comparative Study

**DOI:** 10.3390/antiox13091097

**Published:** 2024-09-10

**Authors:** Vincenzo Disca, Fabiano Travaglia, Chiara Carini, Jean Daniel Coïsson, Giancarlo Cravotto, Marco Arlorio, Monica Locatelli

**Affiliations:** 1 Department of Pharmaceutical Sciences, Università del Piemonte Orientale, Largo Donegani 2, 28100 Novara, Italy; vincenzo.disca@uniupo.it (V.D.); chiara.carini@uniupo.it (C.C.); jeandaniel.coisson@uniupo.it (J.D.C.); marco.arlorio@uniupo.it (M.A.); monica.locatelli@uniupo.it (M.L.); 2 Department of Drug Science and Technology, University of Turin, Via P. Giuria 9, 10125 Turin, Italy; giancarlo.cravotto@unito.it

**Keywords:** *Theobroma cacao*, cocoa bean shells, by-product valorization, polyphenols, microwave-assisted extraction, ultrasound-assisted extraction

## Abstract

The extraction of bioactive compounds from food by-products is one of the most important research areas for the nutraceutical, pharmaceutical, and food industries. This research aimed to evaluate the efficiency of Ultrasound-Assisted Extraction (UAE) and Microwave-Assisted Extraction (MAE), either alone or in combination, of phenolic compounds from cocoa bean shells (CBSs). These extraction techniques were compared with conventional methods, such as under simple magnetic stirring and the Soxhlet apparatus. After the preliminary characterization of the gross composition of CBSs, the total polyphenol content and radical scavenging of extracts obtained from both raw and defatted cocoa bean shells were investigated. Quantification of the main polyphenolic compounds was then performed by RP-HPLC-DAD, identifying flavonoids and phenolic acids, as well as clovamide. The application of MAE and UAE resulted in a similar or superior extraction of polyphenols when compared with traditional methods; the concentration of individual polyphenols was variously influenced by the extraction methods employed. Combining MAE and UAE at 90 °C yielded the highest antiradical activity of the extract. Spectrophotometric analysis confirmed the presence of high-molecular-weight melanoidins, which were present in higher concentrations in the extracts obtained using MAE and UAE, especially starting from raw material. In conclusion, these results emphasize the efficiency of MAE and UAE techniques in obtaining polyphenol-rich extracts from CBS and confirm this cocoa by-product as a valuable biomass for the recovery of antioxidant compounds, with a view to possible industrial scale-up.

## 1. Introduction

Cocoa (*Theobroma cacao* L.) beans and cocoa-derived products have established their prominence in the global market, prized for their delightful flavor and continuously escalating demand [1]. Beyond their reputation as delectable food ingredients, cocoa holds by-products and waste potentially useful as a source of bioactive compounds, particularly phenolics, and fibers, with applications in food, nutraceuticals, pharmaceuticals, and cosmetics [2].

Originating from tropical regions like Ivory Coast, Ecuador, and Nigeria, as well as other regions from Asia, with a gross world production estimated in 2021 at 5,580 million tonnes [3], cocoa fruits are characterized by a complex structure, with seeds embedded within a mucilaginous pulp and a protective coriaceous pod, called *cabosse* [4,5]. Additionally, the seeds harbor a fatty heart, enclosed by a fibrous shell, commonly referred to as cocoa bean shells (CBSs) or cocoa hulls, which are easily separated during the roasting process, after the winnowing process [2,6,7]. Astonishingly, the production of just one kilogram of cocoa powder generates a substantial ten kilograms of waste, mainly composed by the external pod (rich in fiber, polyphenols, and carotenoids) and the mucilaginous pulp during on-farm production, as well as the cocoa shells that are generated during the roasting phase [8]. While dealing with the wasted mucilaginous pulp and cabosse poses challenges, CBSs offer an opportunity for valorization and exploitation, aligning with the principles of a circular economy [9,10]. The concept of upcycling food waste and by-products is a key solution in a circular economy. Upcycling can be defined as a value-enhancing use of ingredients/by-products otherwise wasted—that leads to upcycled food (or upcycled new ingredients). Mainstreaming the idea of upcycling in food systems has huge potential for improving circularity in the food system. The application of green, sustainable, and energy-saving approaches could upcycle many food-generated sources, leading to new products [11] that often relapse in the “novel foods” definition, in accordance with Regulation (EU) 2015/2283 [12].

Moreover, the recovery of bioactive substances is also a key potential topic in the pharmaceutical and nutraceutical fields [13].

In fact, besides the presence of theobromine, caffeine, and a residual fat fraction, CBSs are rich in various bioactive compounds, prominently including polyphenols and brown-pigmented melanoidins (from the Maillard reaction), known for their potent radical-scavenging properties, making them valuable functional ingredients [14].

Traditional organic solvent-based extraction methods have been widely used in the past to harness these compounds from roasted CBSs. However, concerns about cost-effectiveness, environmental sustainability, and efficiency have prompted the exploration of more eco-friendly alternatives [15,16,17].

Ultrasound-Assisted Extraction (UAE) and Microwave-Assisted Extraction (MAE) have emerged as efficient techniques, allowing for intensified extraction and significantly reducing the extraction times, but also improving yields and often enhancing extract quality [18,19,20]. Both techniques have found applications in laboratories, with potential for industrial-scale extraction [21]. Moreover, combining UAE and MAE has shown promising results in extracting bioactive compounds from various sources [22]. The application of UAE and MAE fits well with the concept of extraction intensification [23].

In view of these advances, the present study aims to leverage these non-conventional methods to optimize and intensify the extraction of antioxidant compounds (polyphenols and melanoidins) from CBSs, supporting a circular economy with more efficient processes. Indeed, in this work, we evaluated the efficiency of UAE and MAE both alone and in combination, utilizing two different operative temperatures (50 and 90 °C), and compared them to conventional techniques such as the use of the solvent under agitation (magnetic stirring) and continuous extraction utilizing the Soxhlet apparatus. By applying these innovative techniques, academia and industries contribute to the development of a more economical and environmentally conscious cocoa industry, by valorizing cocoa by-products and leading to new products ready to be used in different fields as antioxidants, anti-inflammatory compounds, or pigmented brown extracts [2].

## 2. Materials and Methods

### 2.1. Materials

Dichloromethane was purchased from Carlo Erba (Rodano, Milano, Italy). Methanol, acetonitrile (all HPLC grade), and formic acid (50%, LC–MS grade) were purchased from Sigma–Aldrich (Milano, Italy). Water was obtained using a Milli-Q instrument (Millipore Corp., Bedford, MA, USA). All reagents and standard chemicals used for the determination of total phenolic content and antiradical activity and HPLC analysis were purchased from Sigma–Aldrich (Milano, Italy).

### 2.2. Samples

Cocoa bean shells were obtained as a by-product (during the pre-roasting phase of cocoa production) from Ecuadorian cocoa beans (Nacional variety). They were kindly provided by an Italian leader manufacturer (Elah Dufour Novi SpA Group, Novi Ligure, Italy).

### 2.3. Proximate Analysis

Firstly, the raw material was finely minced (particle size < 250 µm) with a ball mill (MM400, Retsch Technology, Haan, Germany). Proximate composition (moisture, ash, fat, protein) was analyzed using AOAC methods as reported elsewhere [24]. Ash content was determined thermogravimetrically, and the moisture content was determined using a Sartorius MA30 thermo-balance (Sartorius AG, Goettingen, Germany). Soxhlet extraction (using Soxhlet Büchi Extraction System B-811) and the Kjeldahl method (using Kjeltec system I, Tecator, Sweden) were carried out to calculate fat and protein content, respectively.

### 2.4. Extraction Conditions

The extraction was carried out from the raw material, referred to as “raw CBS”, and from the defatted matrix, referred to as “defatted CBS”. Defatting was performed by extraction in an automatic apparatus (Soxhlet Büchi Extraction System B-811) for 12 h with dichloromethane. The phenolic fraction was extracted from raw and defatted powders in a 1:10 solid-to-solvent ratio, 5 g (particle size < 250 µm) in 50 mL of acidified methanol (trifluoroacetic acid 0.3%, *v*/*v*). Five different conditions were utilized: magnetic stirring (MS) (60 min, 38 °C); Soxhlet apparatus with a cycle of 7 h (SOX); ultrasound (US) with a probe system (titanium horn) (60 min, 38 °C, 20.6 kHz, 80 W); microwaves (MWs) in a closed vessel (professional oven Microsynth Milestone—Bergamo, Italy) (30 min, 90 °C); simultaneous US + MW irradiation, performed by inserting a horn made of a special PEEK^®^ containing glass fibers (by Danacamerini sas, Turin, Italy) for 60 min from the top of the oven with two different operating temperatures, 50 °C (US/MW 50) and 90 °C (US/MW 90) and constant parameters of 21.5 kHz and 60 W. The extraction solvent was evaporated to dryness in a vacuum at 37 °C. Dry extracts were stored at −20 °C and re-dissolved in LC/MS-grade methanol before all analyses.

### 2.5. Spectrophotometric Assays

#### 2.5.1. Specific Extinction Coefficient at 280, 320, and 420 nm

The specific extinction coefficient (K_mix_) at 280, 320, and 420 nm was used to assess the presence of polyphenols and melanoidins in CBS extracts [25,26]. The absorbance of opportunely diluted extracts (1 mg/mL in methanol) was recorded using a Shimadzu UV-1900 spectrophotometer (Shimadzu, Tokyo, Japan) at 280 nm (polyphenols), 320 nm (soluble pre-melanoidins), and 420 nm (high-molecular-weight brown-colored melanoidins). K_mix_ values at these different wavelengths were then calculated through the Lambert–Beer law as follows:

K_mix_ (mg/mL × cm) = A*_sample_*/concentration

#### 2.5.2. Determination of Total Phenolic Content

Total phenolic content (TPC) determination was performed using the classic Folin–Ciocalteu assay [27]. Folin–Ciocalteu reagent (5 mL) and 10 mL of aqueous Na_2_CO_3_ (10% *w*/*v*) were added to 500 µL of sample solution (5 mg/mL in methanol) in a 25 mL volumetric flask. After incubation at 65 °C for 20 min, the solutions were diluted with water to 25 mL and absorbance was read at 760 nm using a Shimadzu UV-1900 spectrophotometer (Shimadzu, Tokyo, Japan). Results were expressed as catechin equivalents (CE)/g of extract or CBSs, through a calibration curve of (±)-catechin (y = 0.0036× − 0.0332, R^2^ = 0.9911).

#### 2.5.3. Radical-Scavenging Activity

The radical-scavenging activity was measured using the DPPH^•^ assay, as previously reported by Locatelli et al. [28]. Briefly, 700 μL of sample or methanol (blank) was added to 700 μL of 100 μM DPPH^•^ solution and allowed to react while protected from light for 20 min. The absorbance of the solution was then read at 515 nm (Shimadzu UV-1900, Shimadzu, Tokyo, Japan). The absorbance value of the blank should fall within the range of 0.500 ± 0.040. The percentage inhibition of DPPH^•^ was calculated using the following equation:

% inhibition = (A*_blank_* − A*_sample_*)/A*_blank_* × 100

CBS extracts were dissolved in methanol (1 mg/mL) and assayed at different concentrations to construct, for each sample, a calibration curve of concentration vs. percentage inhibition, from which the EC_50_ was calculated by interpolation. At least three antioxidant activity curves were created for each extract. These curves were processed through the probit regression model; the final results were expressed as the mean of at least three independent experiments, accompanied by the 95% confidence intervals. A comparison among different antiradical activity curves (raw vs. defatted samples) was obtained as previously reported [28].

### 2.6. RP-HPLC-DAD Analysis

For the RP-HPLC-DAD analysis, a Shimadzu LC-20A Prominence chromatographic system equipped with a diode array detector (DAD, SPD-M20A) was used; the separation was performed on a reversed-phase Luna C18 (150 × 2 mm, 5 μm) (Phenomenex, Torrance, CA, USA), protected by a guard column containing the same phase, at 27 °C. Eluent A of the mobile phase consisted of water/formic acid (99.9:0.1, *v*/*v*), while eluent B consisted of acetonitrile/formic acid (99.9:0.1, *v*/*v*). The applied program gradient was as follows: from 6 to 10.5% of B for 5 min, from 10.5 to 11% B for 15 min, from 11 to 100% B for 26 min, from 100 to 6% B for 2 min, and 6% B for 5 min, with a total run time of 53 min. The flow rate and the injection volume were 400 μL/min and 3 μL, respectively; chromatograms were recorded at 280, 330, and 420 nm.

CBS extracts were dissolved in HPLC-grade methanol at a concentration of 10 mg/mL, and then centrifuged (14,000 rpm for 20 min, microcentrifuge 5417R, Eppendorf, Milan, Italy) prior to their injection in the chromatographic system. The quantification of individual phenolic compounds was performed based on calibration curves obtained with the corresponding standard compounds (gallic acid, protocatechuic acid, benzoic *p*-OH acid, caffeic acid, catechin, epicatechin, quercetin, and kaempferol were expressed as µg/g of extract and µg/g of CBSs); proanthocyanidin quantification was performed based on previous studies and expressed as mg catechin equivalents (CE)/g of extract and g of CBSs [29].

### 2.7. Statistical Analysis

Statistical analyses were conducted using the open-source software R version 4.1.1 [30]. The results were presented as the mean ± standard deviation (SD) of a minimum of three independent experiments. To assess differences, we utilized analysis of variance (ANOVA) followed by Tukey’s honest significant difference test. A significance level of 0.05 was adopted.

## 3. Results

### 3.1. Proximate Composition

The content of moisture, protein, ashes, and lipids is depicted in Table 1. The moisture and protein contents of CBSs were in a range comparable to data reported in the literature, with percentages going from 4% to 10.1% [7,31] for moisture, from 6.2% to 18.1% for protein content, and from 2.02% to 18.2% for lipids [5,7,32].

### 3.2. Specific Extinction Coefficient at 280, 320, and 420 nm

The specific extinction coefficient at 280, 320, and 420 nm was assessed to approximately evaluate the composition of the different extracts, based on the presence of polyphenols, soluble pre-melanoidins, and high-molecular-weight brown-colored melanoidins. The specific extinction coefficient (K_mix_) was used instead of the molar extinction coefficient (ε) due to the presence of different compounds at different molecular weights; furthermore, the molecular weight of melanoidins is unknown and probably variable. By using K_mix_, the concentration in the Beer–Lambert law is expressed in L g^−1^ cm^−1^, which makes it applicable to cocoa bean shells. In addition, concerning the selected wavelengths, it is known that the absorption peak at 280 nm is due to the presence of phenolic compounds, but also eventual proteins, and similarly the absorption peak at 320 nm can be associated with the presence of phenolic acids and flavonoids. Furthermore, it is well accepted that melanoidins, encompassing conjugated systems, absorb across the entire spectrum, but the measurement of melanoidins at wavelengths from 405 to 420 nm is widely reported [25,26]. In fact, melanoidins have been reported to have a unique absorption signature at 405 and 420 nm, which was suggested to correspond to the core structure (405 nm) and the chromophore groups of melanoidins (420 nm) [26].

Based on these considerations, it can be concluded that absorbance readings at 280, 320, and 420 nm offer valuable insights into the relative quantities of polyphenols and melanoidins in the extracts.

**Figure 1:**
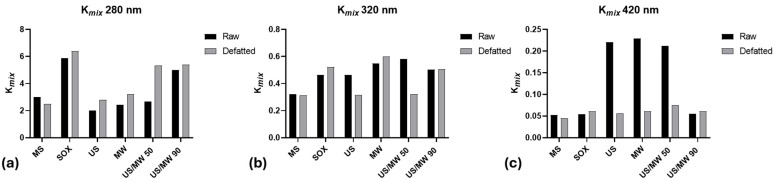
Extinction coefficient K_mix_ at 280 nm (**a**), 320 nm (**b**), and 420 nm (**c**) in the different extracts of CBSs. MS: magnetic stirring; SOX: Soxhlet; US: ultrasound; MW: microwaves; US/MW 50: ultrasound and microwaves at 50 °C; US/MW 90: ultrasound and microwaves at 90 °C.

Figure 1 shows the K_mix_ values for the different extracts, from both raw and defatted CBSs, at 280, 320, and 420 nm. The SOX extraction methods show the highest value at 280 for both raw and defatted shells. These data indicate that the Soxhlet apparatus efficiently extracts substances from the matrix; unfortunately, 280 nm is the wavelength of absorption of the aromatic ring, and thus, most phenolic as well as protein residues absorb it. High values of K_mix_ were also obtained for the sample US/MW 90, both from raw and defatted CBSs, followed by US/MW 50 from defatted CBS. These data show that the unconventional extraction method of MW associated with US at 90 °C was particularly effective in extracting material from the matrix in both raw and defatted shells, but this cannot be associated directly with bioactive phenolic compounds, and more specific analyses are needed.

Concerning melanoidins, the highest values of K_mix_ at 420 nm were obtained for US, MW, and US/MW 50 from raw CBSs. These values suggest that the highest yield of melanoidins is obtained through the unconventional extraction of raw CBSs, whereas the same results are not obtained for defatted CBSs.

### 3.3. Radical-Scavenging Activity

The CBS extracts were characterized for their radical-scavenging activity toward DPPH radicals. Different concentrations were tested to obtain the corresponding activity curves (Supplementary Material Figure S1). In Table 2, the EC_50_ (concentration required to obtain 50% radical-scavenging activity) values and the respective 95% confidence intervals of each extract are reported. EC_50_ is a common parameter for evaluating the antioxidant capacity of the analyzed samples: the lower its value, the greater the antioxidant activity of the extract under consideration.

Among the extracts derived from the raw CBSs, those showing higher antiradical activity are SOX and US/MW 90, with EC_50_ values of 143.97 μg/mL and 163.23 μg/mL, respectively, while other extracts have significantly lower activity, decreasing in the following order: MW ≥ US ≥ MS ≥ US/MW 50.

For the defatted matrix, on the other hand, the extracts with higher values are SOX, US/MW 90, and MW with values of 158.20 μg/mL, 170.22 μg/mL, and 196.29 μg/mL, respectively, followed by US/MW 50 with EC_50_ values of 363.69 μg/mL. The other values do not show significant differences.

Comparing EC_50_ values obtained from raw and defatted CBSs by the same extraction technique, it can be observed that in general, there are no significant differences, except in the case of MW and US/MW 50 treatments, which showed higher antiradical activity in the defatted sample. A more comprehensive evaluation was carried out by comparing the activity curves, for each extraction technique, between raw and defatted CBSs (Figure S2). The curve comparison analysis confirmed better antioxidant properties for the MW and US/MW 50 extracts obtained from defatted CBSs; in addition, a significant difference was also observed for the curves obtained by MS. In this specific case, although the EC_50_ values are similar (raw = 548.74 μg/mL and defatted = 562.28 μg/mL), the curves are significantly different (*p* < 0.001) specifically for the extract obtained from raw CBSs compared to defatted CBSs, which showed higher efficacy in inhibiting the radical at higher concentrations than the EC_50_ value.

### 3.4. Total Phenolic Content

Total phenolic content was assessed by the Folin–Ciocalteu assay; the final results were expressed as mg catechin equivalents for both dry extract and CBSs weights in raw and defatted samples (Table 3). The highest phenolic content in the extracts was observed for samples obtained using MAE, evidencing no significant difference depending on the defatting, followed by the other techniques. While MW extraction was not affected by the matrix pretreatment, the US extraction method showed a higher efficiency for raw CBSs compared to defatted CBSs. The combined use of MW and US on both raw and defatted CBSs did not show a significant positive effect on the TPC of the extracts obtained.

Interestingly, when comparing the effect of different extraction techniques on the TPC with respect to the weight of CBSs, the highest values were observed for US-assisted extraction; in this case, the use of a raw matrix permitted us to obtain the highest polyphenol extraction amount (15 mg CE/g and 6.37 mg CE/g from raw and defatted CBSs, respectively). The same trend is observed for MW-assisted extraction with a higher TPC obtained from raw CBSs (7.16 mg CE/g) compared to defatted CBSs (5.81 mg CE/g).

When looking at the TPC, referred to in grams of CBSs, the conventional extraction techniques MS and SOX were not as performant as the unconventional US and MW techniques for raw CBSs, suggesting that the efficiency is increased by these unconventional techniques, and also that the defatting process can be avoided.

Despite these results, it is worth mentioning that the combination of the two technologies did not show a higher efficiency than the single technologies at either operating temperature (50 and 90 °C), showing that the use of a single technology, simpler from an operational point of view and cheaper in terms of energy consumption, results in a more efficient extraction of phenols from CBSs.

Moreover, the duality of the results, expressed both in terms of TPC per gram of extract and TPC per gram of CBSs, allowed us to highlight the most efficient techniques to obtain both richer functional extracts and higher extraction yields from the matrix itself. However, the limitations of the Folin–Ciocalteu assay must be acknowledged, i.e., the lack of specificity since amino acids and other compounds containing a benzene ring are also detected [33]; for this reason, more specific analyses are needed to further evaluate the effect of the different extraction techniques.

### 3.5. HPLC-DAD Analysis

CBSs were also analyzed by HPLC-DAD for the quantification of the main phenolic compounds. The results given in Table 4 are expressed in grams of extract (mean ± standard deviation) and permit the evaluation of the quality of the extract obtained using the different extraction techniques; instead, the values in Table 5 are expressed as grams of CBSs (mean ± standard deviation) and represent the absolute extraction yield from the initial matrix.

Looking at the extract composition, the most represented compounds are protocatechuic acid and epicatechin. Protocatechuic acid showed the highest concentration in the SOX extract obtained from defatted CBSs (1846 µg/g), followed by the SOX extraction from raw CBSs (1413 µg/g). For the unconventional extraction method, the most efficient was the combination of US and MW at 90 °C (US/MW 90) for raw CBSs with a concentration of 1459 µg/g; a similar value (1453 µg/g) was obtained for defatted CBSs extracted by MW. Regarding the absolute value of protocatechuic extracted from CBSs, the highest yield was obtained from raw CBSs extracted with US, yielding 225 µg/g, while the Soxhlet (SOX) method resulted in 158 µg/g, and the combination of US and MW at 90 °C (US/MW 90) produced 151 µg/g. For defatted CBSs, the highest concentration was observed with US at 196 µg/g, followed by MW at 168 µg/g and SOX at 162 µg/g.

Epicatechin, the most abundant flavan-3-ol in cocoa and cocoa-derived products [8,34], was shown to be particularly present in the extract obtained through conventional SOX extraction with defatted CBSs (1640 µg/g), significantly higher than that extracted from raw CBSs (1250 µg/g). While SOX extraction was particularly efficient for defatted CBSs, unconventional US/MW 90 extraction was shown to be performant for both raw (1500 µg/g) and defatted CBSs (1520 µg/g), followed by MW extraction for defatted CBSs (1240 µg/g). The same trend is confirmed for absolute extraction from CBSs (Table 5), where epicatechin showed the highest concentration when extracted with US/MW 90, yielding 155 µg/g for raw CBSs and 130 µg/g for defatted CBSs. Extraction through SOX resulted in 140 µg/g for raw CBSs and 144 µg/g for defatted CBSs. Instead, magnetic stirring (MS) yielded a very low quantity, compared to the unconventional methods and the time-consuming SOX, with 68.4 µg/g for raw CBSs and 70.1 µg/g for defatted CBSs.

Clovamide, a very strong antioxidant compound characteristic of cocoa and cocoa-derived products [27], was higher in extracts obtained through the unconventional combination of US/MW 90, both in raw CBSs (139 µg/g) and defatted CBSs (140 µg/g). SOX extraction was less efficient in obtaining extracts rich in clovamide, with 116 µg/g from raw CBSs and 134 µg/g from defatted CBSs. Neither extraction showed significant differences between raw and defatted CBSs. When projecting the results to the absolute amount recovered from CBSs, clovamide was extracted more efficiently from raw CBSs through US, resulting in 16.8 µg/g, while US/MW 90 extraction yielded 14.4 µg/g from raw CBSs and 12.0 µg/g from defatted CBSs. Conventional SOX extraction yielded 13.0 µg/g from raw CBSs and 11.7 µg/g from defatted CBSs.

Contradictory to this is the behavior of gallic acid, which showed very high concentrations for some of the extracts, in particular from raw CBSs by the US/MW 90 method with 1050 µg/g, but was quite low for extracts obtained by US and MW, especially from defatted CBSs. Peculiarly, when gallic acid was poorly extracted, we obtained a higher content of caffeic acid. Both compounds showed the same trend in the total amount recovered from CBSs (Table 5) with the highest amount obtained through the unconventional US/MW 90 method and by extraction through US.

Looking at catechin (Table 4), it is possible to appreciate that is particularly present in the extract obtained by the conventional SOX method from defatted CBSs (139 µg/g), significantly higher than the extract obtained from raw CBSs (93.9 µg/g). Notably, using the unconventional US/MW 90 method on raw CBSs yielded an extract comparable to the one obtained through SOX with a value of 94.2 µg/g. When looking at the total amount obtained from CBSs (Table 5), catechin was most abundantly extracted from defatted CBSs using SOX (12.2 µg/g) compared to raw CBSs (10.5 µg/g), while extraction through US/MW 90 produced 9.71 µg/g from raw CBSs, and MS at 3.20 µg/g.

Kaempferol and quercetin are the two least abundant flavonoids quantified in the extract (Table 4). Quercetin showed the highest values in the extract obtained through MW (68.9 µg/g) from defatted CBSs, followed by US/MW 90 from both defatted and raw CBSs (53.8 µg/g and 38.9 µg/g, respectively, with no significant difference depending on defatting). On the contrary, kaempferol showed the highest concentration in the extract obtained through the conventional SOX methodology on raw CBSs (18.9 µg/g), significantly higher compared to the defatted CBSs (10.9 µg/g). The amount of MW-derived extract was lower than that obtained through SOX and MS, but was significant compared to the other methods. Looking at the total amount recovered from CBSs (Table 5), kaempferol showed the highest concentration in raw CBSs extracted with SOX, yielding 2.11 µg/g, while MW resulted in 1.35 µg/g, and US in 1.26 µg/g. In contrast, quercetin was most abundantly extracted from defatted CBSs through MW (7.98 µg/g) followed by US/MW 90 with 4.62 µg/g from defatted CBSs and 4.02 µg/g from raw CBSs.

Finally, proanthocyanidins were identified as reported in previous work and quantified as mg CE/g. The extract that was most abundant in this class of compounds was obtained through the unconventional MW technique, with 28.7 mg/g from raw CBSs, significantly higher than 27.3 mg/g from defatted CBSs. This was followed by the extract obtained through the unconventional US/MW 90 technique with a value of 27.9 mg/g for defatted CBSs, higher than the 24.8 mg/g obtained from raw CBSs. The conventional methodologies (MS and SOX) show less efficacy in extracting proanthocyanidins from CBSs.

If looking at the total amount of these molecules extracted from CBSs, the highest concentration is observed from raw CBSs extracted with US, yielding 8.21 mg/g (significantly higher than 4.68 mg/g from defatted CBSs), while MW resulted in 4.11 mg/g from raw CBSs and 3.17 mg/g from defatted CBSs. Conventional extraction techniques and the combination of unconventional technologies resulted in a lower amount of proanthocyanidins being recovered from CBSs.

## 4. Discussion

Cocoa bean shells (CBSs) are a by-product of the cocoa industry and are an important source of bioactive compounds, including polyphenols, mainly flavonoids and methylxanthines, which are known for their antioxidant, anti-inflammatory, and potential therapeutic properties [35]; they also contain dietary fiber with potential prebiotic properties [36]. The particularly high content of polyphenols makes CBSs a feasible and inexpensive upcycled source of antioxidant compounds for the nutraceutical, pharmaceutical, and food industries. Polyphenols such as catechins, epicatechins, and proanthocyanidins are abundant in CBSs and contribute significantly to their antioxidant capacity. In addition, clovamide, a derivative of caffeic acid and an amide isostere of rosmarinic acid known to have potent antioxidant activity, stronger than that of other polyphenols [28], was also recognized in cocoa and cocoa bean shells [27,37]. Furthermore, the presence of melanoidins, formed during the roasting process, also contributes to the antioxidant potential of CBSs [38,39].

The results of this study provide valuable insights into the effectiveness of different techniques in extracting bioactive compounds from CBSs. In particular, the unconventional techniques, namely UAE and MAE, either alone or in combination, were found to be highly efficient in extracting antioxidants compared to conventional methods.

UAE uses ultrasonic waves to create cavitation bubbles in the solvent, which collapse to create microjets that disrupt plant cell walls. This enhances the release of intracellular compounds, making it an efficient method for extracting heat-sensitive bioactive compounds from CBS [40]. UAE has been shown to significantly reduce extraction time and solvent consumption compared to traditional methods while improving extraction yields for compounds such as catechins and epicatechins [41]. MAE uses MW radiation to heat the solvent and plant material, increasing kinetic energy and disrupting cell walls to facilitate the release of bioactive compounds [32]. This technique has the advantage of rapid heating, minimizing the thermal degradation of sensitive compounds, and increasing the extraction efficiency of polyphenols and other antioxidants.

In this work, we tested different extraction conditions. Regarding the extraction duration, the conventional methods were set to 60 min for MS and 7 h for SOX. For unconventional US, US/MW 50, and US/MW 90, the duration was set to 60 min, whereas MW-assisted extraction was set to a shorter duration of 30 min. This implies that Soxhlet extraction is the most time-consuming procedure, while MW extraction is the shortest, operating at the highest temperature of 90 °C, along with US/MW 90.

Among the unconventional techniques, the combination of US and MW turned out to be the best in terms of antiradical activity of the extract obtained from raw and defatted CBSs, comparable in terms of EC_50_ values to the traditional method with the Soxhlet apparatus. In particular, the US/MW combination is significantly more effective at 90 °C than at 50 °C for radical-scavenging activity, a difference that is not statistically significant for the TPC of the extract from both raw and defatted matrices.

The ultrasound combined with MW at 90 °C (US/MW 90) technique showed the highest antiradical activity despite a lower total polyphenolic content. This suggests that the antioxidant activity may be influenced more by the types of polyphenols extracted than by their total amount. Locatelli et al. highlighted that specific phenolic compounds have higher antioxidant activity and can be selectively extracted using unconventional technologies [28].

The higher efficiency in the extraction of specific antioxidants, such as catechin and epicatechin, by UAE and MAE was confirmed by the observations of Chemat et al., who reported that these techniques improve the extraction of heat-sensitive and hydrophobic compounds, due to the employment of controlled temperature and the use of solvents [18]. Moreover, the high level of flavan-3-ols, mainly epicatechin and catechin, and proanthocyanidins in the SOX and US/MW 90 extractions explains the high antioxidant activity in CBS extracts treated with these techniques. This is in line with what has been shown by Cádiz-Gurrea et al. (2020), who, by a correlation study between radical-scavenging activity and chemical structure, demonstrated that these classes of bioactive compounds are powerful antioxidants due to their electronic delocalization [42].

Regarding the total polyphenolic content, innovative treatments are better than conventional ones; in particular, MAE shows the highest TPC values on both raw and defatted extracts (50.1 and 53.2 mg CE/g extract, respectively). This was confirmed by Mellinas et al. (2020), who showed that MW is more effective than conventional methods, such as Soxhlet extraction [32]. In fact, the high temperature reached by MW and US/MW 90 could help to improve the extraction of phenolic compounds. The advantage of US/MW 90 is the combination of the two technologies; on the other hand, the MW extraction was carried out in less time. However, both techniques underline the efficiency of extraction in a short time compared to continuous and time-consuming SOX extraction.

The characterization of phenolic compounds by RP-HPLC revealed a higher content of proanthocyanidin compounds in extracts obtained by innovative techniques compared to conventional methods. This confirms the efficiency of UAE and MAE in preserving or extracting specific bioactive compounds that contribute significantly to antioxidant properties. In line with the observations of Bouchez et al. (2023), the higher yields of proanthocyanidins can be attributed to rapid heating in MAE, which facilitates the release of bound polyphenols from the CBS matrix [43]. In fact, the MW extraction yielded the highest proanthocyanidin content for both raw and defatted CBSs, followed by the US/MW 90 extraction method, which can highlight the importance of the temperature used to assist the extraction, more specifically for defatted CBSs. Interestingly, the extraction technique using US/MW 90 showed very similar results between raw and defatted CBSs, demonstrating the potential for industrial-scale application without the need for a prior lipid extraction step. This could reduce processing time and cost, making the process more efficient and sustainable. The work of Mariatti et al. (2021) confirmed that combined extraction techniques are effective in different matrices, reducing the need for extensive pre-processing [44].

Among non-conventional extraction techniques, US, MW, and US/MW 50 were particularly effective in extracting melanoidins from raw CBSs, presenting the highest K_mix_ values at 420 nm. In the literature, it is reported that melanoidins, which are high-molecular-weight compounds formed during the Maillard reaction, have strong antioxidant properties, and their efficient extraction is beneficial for obtaining high-quality antioxidant extracts [25]. However, considering that these extracts did not present higher antioxidant properties compared to the respective extracts obtained from defatted CBSs, characterized by lower melanoidin content, it can be hypothesized that the contribution of melanoidins to the total antioxidant activity of the extract is not as influent as that of polyphenols.

Moreover, it is worth noting that, looking at the absolute amount recovered from CBSs for specific compounds such as protocatechuic acid, *p*-OH benzoic acid, caffeic acid, and most notably clovamide, the US extraction alone from raw CBSs was shown to be significantly higher than all the other extractions. This is in line with a recent work by Ramos Escudero and colleagues, who obtained high-value extracts (rich in antioxidant compounds) with sonotrode-based extraction compared to conventional extraction procedures [41].

While the non-conventional extraction methods showed promising results, it is important to note that the Soxhlet extraction method also showed high efficiency in extracting certain compounds such as catechin and epicatechin. This suggests that while innovative methods offer significant advantages in terms of speed and potential energy savings, traditional methods still have value for certain types of extractions. This is consistent with observations reported in the literature that conventional extraction methods, although more time-consuming, are sometimes more effective for certain compounds due to longer contact time with the solvent and higher extraction efficiency for some polyphenols [45]. In addition, the variability in extraction efficiency for different compounds suggests that a combination of methods may be optimal for the comprehensive extraction of all desired bioactive compounds [46]. As shown in the present work, temperature plays a key role in assisting the extraction of bioactive compounds from CBSs. While a high temperature allows the extraction yield to be increased, too high a temperature may lead to the degradation of the main bioactive compounds in a detrimental manner. In this study, a threshold temperature of 90 °C was set to avoid any possible degradation of the major compounds. Future studies could investigate the effect of higher temperatures on the phenolic fraction using these unconventional techniques.

Finally, defatting CBSs prior to extraction only under certain conditions affected the quality of extracts and the recovery of compounds. This implies that the pretreatment of CBSs by defatting could be unnecessary when considering the potential scale-up of the process. This approach aligns with the concept of a circular economy for waste reduction.

## 5. Conclusions

The objective of this research was to assess the performance of non-conventional extraction techniques using US and MW, and to compare them with conventional extractions (simple magnetic stirring and Soxhlet apparatus) from cocoa bean shells. Our main goal was the recovery of antioxidant compounds for potential pharmaceutical and nutraceutical applications. Among the innovative extraction techniques, the one combining US and MW at 90 °C (US/MW 90) achieved the highest antiradical activity. In fact, this study showed that the combination of UAE and MAE, in particular US/MW 90, was highly effective in extracting a wide range of bioactive compounds from CBSs, including melanoidins, polyphenols, and proanthocyanidins. This suggests that the combination of UAE and MAE at high temperatures can lead to a synergistic effect that further enhances extraction efficiency. Moreover, US-assisted extraction alone resulted in a very high recovery of bioactive compounds from the starting matrix. This would suggest that a “tailored” approach should be considered both to obtain a rich antioxidant extract and to obtain high yields of target molecules from CBSs.

Overall, defatted and raw CBSs showed very similar results, except for specific conditions, demonstrating the potential for industrial scale-up without the need for a preliminary lipid extraction step, thus reducing processing time and cost. Future studies could investigate the effect of higher temperatures on the extraction of the main phenolics, possibly using a mathematical model to better describe the influence of the different variables. This study emphasizes how innovative extraction techniques offer the possibility of obtaining bioactive phytochemicals from matrices such as cocoa processing by-products, opening up promising opportunities for their application in the pharmaceutical and nutraceutical industries.

## Figures and Tables

**Table 1:** Proximate composition of cocoa bean shells expressed as percentages.

Components	Value (%)
Moisture	9.37 ± 0.11
Protein	16.33 ± 0.07
Ashes	9.34 ± 0.09
Lipids	3.34 ± 0.33

**Table 2:** EC_50_ value, expressed as μg/mL, for the inhibition of DPPH radicals, obtained for the different CBS extracts; different lowercase letters in the same column indicate significantly different samples (*p* < 0.05). For the same extraction technique, if present, different capital letters indicate significantly different samples between raw and defatted CBSs (*p* < 0.05).

	EC_50_ ÷ (CI 95%)	
	Raw	Defatted
MS	548.74 (491.70 ÷ 612.07) ^bc^	562.28 (497.33 ÷ 635.77) ^c^
SOX	143.97 (127.83 ÷ 162.08) ^a^	158.20 (146.82 ÷ 170.42) ^a^
US	538.57 (511.58 ÷ 567.04) ^bc^	622.65 (549.49 ÷ 705.69) ^c^
MW	481.07 (423.79 ÷ 545.89) ^bB^	196.29 (175.82 ÷ 219.13) ^aA^
US/MW 50	614.17 (587.90 ÷ 641.64) ^cB^	363.69 (326.92 ÷ 404.42) ^bA^
US/MW 90	163.23 (151.01 ÷ 176.40) ^a^	170.22 (156.65 ÷ 184.91) ^a^

MS, magnetic stirring; SOX, Soxhlet; US, ultrasound; MW, microwaves; US/MW 50, ultrasound and microwaves at 50 °C; US/MW 90, ultrasound and microwaves at 90 °C.

**Table 3:** Total phenolic content (mg CE/g) expressed as mean ± standard deviation. Values with different lowercase letters in the same column are significantly different (*p* < 0.05). For the same extraction technique and within the same TPC group, different capital letters, if present, indicate significantly different samples (*p* < 0.05).

	TPC (mg CE/g Extract)		TPC (mg/CE g CBSs)	
	Raw	Defatted	Raw	Defatted
MS	41.5 ± 4.1 ^b^	35.3 ± 3.2 ^b^	4.53 ± 0.45 ^c^	3.66 ± 0.34 ^b^
SOX	31.7 ± 1.0 ^cB^	36.6 ± 1.2 ^bA^	3.54 ± 0.11 ^d^	3.12 ± 0.10 ^b^
US	41.2 ± 0.3 ^bA^	34.5 ± 2.2 ^bB^	15.0 ± 0.1 ^aA^	6.37 ± 0.41 ^aB^
MW	50.1 ± 4.3 ^a^	53.2 ± 4.1 ^a^	7.16 ± 0.62 ^bA^	5.81 ± 0.45 ^aB^
US/MW 50	37.0 ± 1.9 ^bc^	37.1 ± 2.4 ^b^	3.53 ± 0.18 ^dA^	2.89 ± 0.19 ^bB^
US/MW 90	38.8 ± 0.3 ^bcA^	38.0 ± 0.3 ^bB^	4.01 ± 0.03 ^cdA^	3.16 ± 0.02 ^bB^

MS, magnetic stirring; SOX, Soxhlet; US: ultrasound; MW: microwaves; US/MW 50: ultrasound and microwaves at 50 °C; US/MW 90: ultrasound and microwaves at 90 °C.

**Table 4:** Individual phenolics quantified through HPLC-DAD expressed as mean ± standard deviation of g of extract. Values with different lowercase letters in the same row are significantly different (*p* < 0.05). For the same extraction technique and the same compound, values with different capital letters, if present, are significantly different (*p* < 0.05).

		MS	SOX	US	MW	US/MW 50	US/MW 90
Protocatechuic acid (µg/g)	Raw	647 ± 4 ^bB^	1413 ± 38 ^aB^	617 ± 10 ^bB^	678 ± 24 ^bB^	623 ± 21 ^bB^	1459 ± 49 ^aA^
	Defatted	869 ± 37 ^dA^	1846 ± 10 ^aA^	1001 ± 10 ^cA^	1453 ± 23 ^bA^	974 ± 12 ^cA^	1008 ± 9 ^cB^
Caffeic acid (µg/g)	Raw	91.0 ± 3.0 ^cB^	155 ± 2 ^bB^	72.6 ± 1.2 ^dB^	58.8 ± 0.4 ^eB^	53.0 ± 0.2 ^eB^	197 ± 7 ^aA^
	Defatted	106 ± 4 ^eA^	211 ± 4 ^aA^	155 ± 3 ^cdA^	186 ± 1 ^bA^	142 ± 2 ^dA^	162 ± 10 ^cB^
*p*-OH benzoic acid (µg/g)	Raw	54.5 ± 2.9 ^dB^	91.8 ± 1.3 ^bB^	68.6 ± 2.6 ^cB^	69.7 ± 7.8 ^cB^	69.1 ± 4.0 ^c^	240 ± 5 ^aA^
	Defatted	77.4 ± 8.9 ^cA^	160 ± 6 ^bA^	86.1 ± 6.5 ^cA^	214 ± 8 ^aA^	79.3 ± 7.3 ^c^	73.8 ± 2.2 ^cB^
Gallic acid (µg/g)	Raw	306 ± 4 ^cA^	462 ± 26 ^bB^	177 ± 1 ^dA^	159 ± 2 ^dB^	150 ± 2 ^dA^	1050 ± 20 ^aA^
	Defatted	298 ± 1 ^cB^	609 ± 15 ^aA^	25,2 ± 2 ^dB^	192 ± 18 ^eA^	24,2 ± 1 ^eB^	360 ± 12 ^bB^
Epicatechin (µg/g)	Raw	627 ± 6 ^c^	1250 ± 10 ^bB^	84.1 ± 3 ^d^	104 ± 13 ^dB^	84.4 ± 6 ^dB^	1500 ± 20 ^a^
	Defatted	637 ± 39 ^d^	1640 ± 20 ^aA^	95.7 ± 8 ^f^	1240 ± 20 ^cA^	414± 23 ^eA^	1520 ± 30 ^b^
Catechin (µg/g)	Raw	29.3 ± 1.9 ^bB^	94.2 ± 4.2 ^aB^	7.16 ± 1.3 ^c^	3.38 ± 0.05 ^cB^	2.36 ± 0.13 ^cB^	93.9 ± 12.9 ^a^
	Defatted	40.1 ± 0.8 ^dA^	139 ± 6 ^aA^	9.35 ± 0.26 ^e^	63.0 ± 7.4 ^cA^	15.8 ± 0.78 ^eA^	79.3 ± 1.1 ^b^
Clovamide (µg/g)	Raw	34.2 ± 4.2 ^cB^	116 ± 13 ^a^	45.9 ± 2.5 ^bc^	56.1 ± 8.6 ^bcB^	57.9 ± 5.5 ^b^	139 ± 4 ^a^
	Defatted	72.5 ± 11.4 ^bcA^	134 ± 9 ^a^	47.1 ± 2.7 ^c^	114 ± 7 ^aA^	75.4 ± 5.1 ^b^	140 ± 13 ^a^
Kaempferol (µg/g)	Raw	4.86 ± 0.15 ^cB^	18.9 ± 1.2 ^aA^	3.46 ± 0.25 ^cB^	9.44 ± 1.21 ^b^	8.68 ± 0.18 ^bA^	7.42 ± 0.12 ^bA^
	Defatted	12.0 ± 1.1 ^aA^	10.9 ± 0.7 ^abB^	4.89 ± 0.08 ^cA^	9.51 ± 0.54 ^b^	5.04 ± 0.37 ^cB^	6.15 ± 0.06 ^cB^
Quercetin (µg/g)	Raw	7.46 ± 0.91 ^cB^	22.4 ± 1.8 ^b^	5.11 ± 0.36 ^c^	24.0 ± 0.09 ^bB^	10.7 ± 1.1 ^cA^	38.9 ± 7.3 ^a^
	Defatted	11.5 ± 0.2 ^dA^	19.1 ± 3.4 ^c^	6.83 ± 1.51 ^de^	68.9 ± 2.1 ^aA^	4.75 ± 0.02 ^eB^	53.8 ± 3.8 ^b^
Proanthocyanidins (mg/g)	Raw	19.2 ± 0.6 ^e^	13.6 ± 0.5 ^fB^	22.5 ± 0.3 ^c^	28.7 ± 0.7 ^aA^	20.6 ± 0.2 ^dB^	24.8 ± 0.1 ^bB^
	Defatted	20.0 ± 0.4 ^d^	19.1 ± 0.2 ^dA^	23.9 ± 0.9 ^b^	27.3 ± 0.2 ^aB^	21.5 ± 0.2 ^cA^	27.9 ± 0.8 ^aA^

MS, magnetic stirring; SOX, Soxhlet; US, ultrasound; MW, microwaves; US/MW 50, ultrasound and microwaves at 50 °C; US/MW 90, ultrasound and microwaves at 90 °C.

**Table 5:** Individual phenolics quantified through HPLC-DAD expressed as mean ± standard deviation of g of CBSs. Values with different lowercase letters in the same row are significantly different (*p* < 0.05). For the same extraction technique and the same compound, values with different capital letters, if present, are significantly different (*p* < 0.05).

		MS	SOX	US	MW	US/MW 50	US/MW 90
Protocatechuic acid (µg/g)	Raw	70.6 ± 0.4 ^dB^	158 ± 4 ^b^	225 ± 4 ^aA^	96.9 ± 3.5 ^cB^	59.4 ± 2.0 ^eB^	151 ± 5 ^bA^
	Defatted	95.7 ± 4.1 ^cA^	162 ± 0.8 ^b^	196 ± 2 ^aB^	168 ± 3 ^bA^	80.6 ± 1.0 ^dA^	86.5 ± 0.8 ^dB^
Caffeic acid (µg/g)	Raw	9.92 ± 0.33 ^dB^	17.3 ± 0.2 ^cB^	26.5 ± 0.4 ^aB^	8.41 ± 0.06 ^eB^	5.05 ± 0.02 ^fB^	20.3 ± 0.7 ^bA^
	Defatted	11.6 ± 0.4 ^eA^	18.5 ± 0.3 ^cA^	30.3 ± 0.6 ^aA^	21.5 ± 0.1 ^bA^	11.8 ± 0.2 ^eA^	13.9 ± 0.9 ^dB^
*p*-OH benzoic acid (µg/g)	Raw	5.95 ± 0.32 ^cB^	10.2 ± 0.2 ^b^	25.0 ± 0.9 ^aA^	10.0 ± 1.1 ^bB^	6.59 ± 0.38 ^c^	24.9 ± 0.53 ^aA^
	Defatted	8.52 ± 0.98 ^dA^	14.0 ± 0.5 ^c^	16.9 ± 1.3 ^bB^	24.8 ± 1.0 ^aA^	6.56 ± 0.60 ^d^	6.34 ± 0.19 ^dB^
Gallic acid (µg/g)	Raw	33.4 ± 0.4 ^d^	51.6 ± 2.9 ^c^	64.6 ± 0.3 ^bA^	22.7 ± 0.2 ^e^	14.3 ± 0.2 ^fA^	108 ± 2 ^aA^
	Defatted	32.7 ± 0.2 ^b^	53.5 ± 1.3 ^a^	4.93 ± 0.46 ^dB^	22.2 ± 2.1 ^c^	2.00 ± 0.06 ^dB^	30.9 ± 1.0 ^bB^
Epicatechin (µg/g)	Raw	68.4 ± 0.6 ^c^	140 ± 1 ^bB^	30.7 ± 1.1 ^dA^	14.8 ± 1.8 ^eB^	8.05 ± 0.54 ^fB^	155 ± 2 ^aA^
	Defatted	70.1 ± 4.2 ^c^	144 ± 2 ^aA^	18.7 ± 1.6 ^eB^	143 ± 3 ^aA^	34.2 ± 2.0 ^dA^	130 ± 3 ^bB^
Catechin (µg/g)	Raw	3.20 ± 0.21 ^bB^	10.5 ± 0.47 ^aB^	2.61 ± 0.47 ^b^	0.484 ± 0.007 ^cB^	0.225 ± 0.013 ^cB^	9.71 ± 1.33 ^aA^
	Defatted	4.41 ± 0.09 ^cA^	12.2 ± 0.55 ^aA^	1.83 ± 0.05 ^d^	7.29 ± 0.86 ^bA^	1.31 ± 0.06 ^dA^	6.81 ± 0.09 ^bB^
Clovamide (µg/g)	Raw	3.73 ± 0.46 ^dB^	13.0 ± 1.4 ^b^	16.8 ± 0.9 ^a^	8.02 ± 1.23 ^cB^	5.52 ± 0.52 ^cd^	14.4 ± 0.47 ^abA^
	Defatted	7.97 ± 1.25 ^cdA^	11.7 ± 0.8 ^ab^	9.22 ± 0.53 ^bc^	13.1 ± 0.76 ^aA^	6.24 ± 0.42 ^d^	12.0 ± 1.1 ^aB^
Kaempferol (µg/g)	Raw	0.530 ± 0.016 ^cB^	2.11 ± 0.14 ^aA^	1.26 ± 0.09 ^bA^	1.35 ± 0.17 ^b^	0.827 ± 0.017 ^cA^	0.767 ± 0.012 ^cA^
	Defatted	1.32 ± 0.12 ^aA^	0.955 ± 0.059 ^bB^	0.957 ± 0.015 ^bB^	1.10 ± 0.06 ^b^	0.417 ± 0.031 ^cB^	0.528 ± 0.005 ^cB^
Quercetin (µg/g)	Raw	0.814 ± 0.099 ^dB^	2.50 ± 0.20 ^bcA^	1.87 ± 0.13 ^cd^	3.43 ± 0.01 ^abB^	1.02 ± 0.10 ^dA^	4.02 ± 0.76 ^a^
	Defatted	1.27 ± 0.02 ^cA^	1.68 ± 0.29 ^cB^	1.34 ± 0.30 ^c^	7.98 ± 0.24 ^aA^	0.393 ± 0.002 ^dB^	4.62 ± 0.27 ^b^
Proanthocyanidins (mg/g)	Raw	2.10 ± 0.07 ^d^	1.51 ± 0.05 ^eB^	8.21 ± 0.11 ^aA^	4.11 ± 0.10 ^bA^	1.96 ± 0.01 ^dA^	2.57 ± 0.01 ^cA^
	Defatted	2.20 ± 0.04 ^c^	1.68± 0.02 ^dA^	4.68 ± 0.17 ^aB^	3.17 ± 0.02 ^bB^	1.78 ± 0.01 ^dB^	2.39 ± 0.07 ^cB^

MS, magnetic stirring; SOX, Soxhlet; US, ultrasound; MW, microwaves; US/MW 50, ultrasound and microwaves at 50 °C; US/MW 90, ultrasound and microwaves at 90 °C.

## Data Availability

Data is contained within the article or Supplementary Material.

## Supplementary Materials

The following supporting information can be downloaded at https://www.mdpi.com/article/10.3390/antiox13091097/s1. Figure S1. Probit regression curves for the different CBSs extracts. Figure S2. Comparison between the activity curves obtained from raw and those obtained for defatted CBS; curves characterized by *p*-values < 0.05 were considered significantly different.

## References

[1] Barišić V., Icyer N.C., Akyil S., Toker O.S., Flanjak I., Ačkar Đ. (2023). Cocoa Based Beverages—Composition, Nutritional Value, Processing, Quality Problems and New Perspectives. Trends Food Sci. Technol..

[2] Rojo-Poveda O., Barbosa-Pereira L., Zeppa G., Stévigny C. (2020). Cocoa Bean Shell—A By-Product with Nutritional Properties and Biofunctional Potential. Nutrients.

[3] FAOSTAT. https://www.fao.org/faostat/en/#data/QCL.

[4] Belwal T., Cravotto C., Ramola S., Thakur M., Chemat F., Cravotto G. (2022). Bioactive Compounds from Cocoa Husk: Extraction, Analysis and Applications in Food Production Chain. Foods.

[5] Vásquez Z.S., de Carvalho Neto D.P., Pereira G.V.M., Vandenberghe L.P.S., de Oliveira P.Z., Tiburcio P.B., Rogez H.L.G., Góes Neto A., Soccol C.R. (2019). Biotechnological Approaches for Cocoa Waste Management: A Review. Waste Manag..

[6] Cinar Z.Ö., Atanassova M., Tumer T.B., Caruso G., Antika G., Sharma S., Sharifi-Rad J., Pezzani R. (2021). Cocoa and Cocoa Bean Shells Role in Human Health: An Updated Review. J. Food Compos. Anal..

[7] Okiyama D.C.G., Navarro S.L.B., Rodrigues C.E.C. (2017). Cocoa Shell and Its Compounds: Applications in the Food Industry. Trends Food Sci. Technol..

[8] Younes A., Li M., Karboune S. (2023). Cocoa Bean Shells: A Review into the Chemical Profile, the Bioactivity and the Biotransformation to Enhance Their Potential Applications in Foods. Crit. Rev. Food Sci. Nutr..

[9] Borrello M., Caracciolo F., Lombardi A., Pascucci S., Cembalo L. (2017). Consumers’ Perspective on Circular Economy Strategy for Reducing Food Waste. Sustainability.

[10] Slorach P.C., Jeswani H.K., Cuéllar-Franca R., Azapagic A. (2019). Environmental and Economic Implications of Recovering Resources from Food Waste in a Circular Economy. Sci. Total Environ..

[11] Aschemann-Witzel J., Asioli D., Banovic M., Perito M.A., Peschel A.O., Stancu V. (2023). Defining Upcycled Food: The Dual Role of Upcycling in Reducing Food Loss and Waste. Trends Food Sci. Technol..

[12] Ververis E., Ackerl R., Azzollini D., Colombo P.A., de Sesmaisons A., Dumas C., Fernandez-Dumont A., Ferreira da Costa L., Germini A., Goumperis T. (2020). Novel Foods in the European Union: Scientific Requirements and Challenges of the Risk Assessment Process by the European Food Safety Authority. Food Res. Int..

[13] Roselli V., Pugliese G., Leuci R., Brunetti L., Gambacorta L., Tufarelli V., Piemontese L. (2024). Green Methods to Recover Bioactive Compounds from Food Industry Waste: A Sustainable Practice from the Perspective of the Circular Economy. Molecules.

[14] Sánchez M., Laca A., Laca A., Díaz M. (2023). Cocoa Bean Shell: A By-Product with High Potential for Nutritional and Biotechnological Applications. Antioxidants.

[15] Panja P. (2018). Green Extraction Methods of Food Polyphenols from Vegetable Materials. Curr. Opin. Food Sci..

[16] Picot-Allain C., Mahomoodally M.F., Ak G., Zengin G. (2021). Conventional versus Green Extraction Techniques—A Comparative Perspective. Curr. Opin. Food Sci..

[17] Gonçalves M.L.M.B.B., Maximo G.J. (2023). Circular Economy in the Food Chain: Production, Processing and Waste Management. Circ.Econ.Sust..

[18] Chemat F., Zill-e-Huma, Khan M.K. (2011). Applications of Ultrasound in Food Technology: Processing, Preservation and Extraction. Ultrason. Sonochemistry.

[19] Routray W., Orsat V. (2012). Microwave-Assisted Extraction of Flavonoids: A Review. Food Bioprocess Technol..

[20] Vilkhu K., Mawson R., Simons L., Bates D. (2008). Applications and Opportunities for Ultrasound Assisted Extraction in the Food Industry—A Review. Innov. Food Sci. Emerg. Technol..

[21] Chan C.-H., See T.-Y., Yusoff R., Ngoh G.-C., Kow K.-W. (2017). Extraction of Bioactives from Orthosiphon Stamineus Using Microwave and Ultrasound-Assisted Techniques: Process Optimization and Scale Up. Food Chem..

[22] Garcia-Vaquero M., Ummat V., Tiwari B., Rajauria G. (2020). Exploring Ultrasound, Microwave and Ultrasound–Microwave Assisted Extraction Technologies to Increase the Extraction of Bioactive Compounds and Antioxidants from Brown Macroalgae. Mar. Drugs.

[23] Shirsath S.R., Sonawane S.H., Gogate P.R. (2012). Intensification of Extraction of Natural Products Using Ultrasonic Irradiations—A Review of Current Status. Chem. Eng. Process. Process Intensif..

[24] Djali M., Santasa K., Indiarto R., Subroto E., Fetriyuna F., Lembong E. (2023). Proximate Composition and Bioactive Compounds of Cocoa Bean Shells as a By-Product from Cocoa Industries in Indonesia. Foods.

[25] Bekedam E.K., Schols H.A., van Boekel M.A.J.S., Smit G. (2006). High Molecular Weight Melanoidins from Coffee Brew. J. Agric. Food Chem..

[26] Feng J., Berton-Carabin C.C., Guyot S., Gacel A., Fogliano V., Schroën K. (2023). Coffee Melanoidins as Emulsion Stabilizers. Food Hydrocoll..

[27] Arlorio M., Locatelli M., Travaglia F., Coïsson J.-D., Grosso E.D., Minassi A., Appendino G., Martelli A. (2008). Roasting Impact on the Contents of Clovamide (N-Caffeoyl-L-DOPA) and the Antioxidant Activity of Cocoa Beans (*Theobroma cacao* L.). Food Chem..

[28] Locatelli M., Gindro R., Travaglia F., Coïsson J.-D., Rinaldi M., Arlorio M. (2009). Study of the DPPH-Scavenging Activity: Development of a Free Software for the Correct Interpretation of Data. Food Chem..

[29] Bordiga M., Travaglia F., Locatelli M., Coïsson J.D., Arlorio M. (2011). Characterisation of Polymeric Skin and Seed Proanthocyanidins during Ripening in Six *Vitis vinifera* L. Cv. Food Chem..

[30] R Core Team (2022). R: A Language and Environment for Statistical Computing.

[31] Disca V., Jaouhari Y., Carrà F., Martoccia M., Travaglia F., Locatelli M., Bordiga M., Arlorio M. (2024). Effect of Carbohydrase Treatment on the Dietary Fibers and Bioactive Compounds of Cocoa Bean Shells (CBSs). Foods.

[32] Mellinas A.C., Jiménez A., Garrigós M.C. (2020). Optimization of Microwave-Assisted Extraction of Cocoa Bean Shell Waste and Evaluation of Its Antioxidant, Physicochemical and Functional Properties. LWT.

[33] Amorati R., Valgimigli L. (2015). Advantages and Limitations of Common Testing Methods for Antioxidants. Free Radic. Res..

[34] La Mantia A., Ianni F., Schoubben A., Cespi M., Lisjak K., Guarnaccia D., Sardella R., Blasi P. (2023). Effect of Cocoa Roasting on Chocolate Polyphenols Evolution. Antioxidants.

[35] Botella-Martínez C., Lucas-Gonzalez R., Ballester-Costa C., Pérez-Álvarez J.Á., Fernández-López J., Delgado-Ospina J., Chaves-López C., Viuda-Martos M. (2021). Ghanaian Cocoa (*Theobroma cacao* L.) Bean Shells Coproducts: Effect of Particle Size on Chemical Composition, Bioactive Compound Content and Antioxidant Activity. Agronomy.

[36] Disca V., Capuano E., Arlorio M. (2024). Colonic Fermentation of Enzymatically Treated Cocoa Bean Shells (CBSs) and Short Chain Fatty Acids (SCFAs) Production. LWT.

[37] Kolodziejczyk-Czepas J. (2024). Clovamide and Its Derivatives—Bioactive Components of *Theobroma cacao* and Other Plants in the Context of Human Health. Foods.

[38] Llerena W., Samaniego I., Vallejo C., Arreaga A., Zhunio B., Coronel Z., Quiroz J., Angós I., Carrillo W. (2023). Profile of Bioactive Components of Cocoa (*Theobroma cacao* L.) By-Products from Ecuador and Evaluation of Their Antioxidant Activity. Foods.

[39] Oracz J., Lewandowska U., Owczarek K., Caban M., Rosicka-Kaczmarek J., Żyżelewicz D. (2024). Isolation, Structural Characterization and Biological Activity Evaluation of Melanoidins from Thermally Processed Cocoa Beans, Carob Kibbles and Acorns as Potential Cytotoxic Agents. Food Chem..

[40] Huynh G.H., Van Pham H., Hong Nguyen H.V. (2023). Effects of Enzymatic and Ultrasonic-Assisted Extraction of Bioactive Compounds from Cocoa Bean Shells. Food Meas..

[41] Ramos-Escudero F., Rojas-García A., de la Luz Cádiz-Gurrea M., Segura-Carretero A. (2024). High Potential Extracts from Cocoa Byproducts through Sonotrode Optimal Extraction and a Comprehensive Characterization. Ultrason. Sonochemistry.

[42] de la Luz Cádiz-Gurrea M., Fernández-Ochoa Á., Leyva-Jiménez F.J., Guerrero-Muñoz N., Villegas-Aguilar M.d.C., Pimentel-Moral S., Ramos-Escudero F., Segura-Carretero A. (2020). LC-MS and Spectrophotometric Approaches for Evaluation of Bioactive Compounds from Peru Cocoa By-Products for Commercial Applications. Molecules.

[43] Bouchez A., Vauchel P., Périno S., Dimitrov K. (2023). Multi-Criteria Optimization Including Environmental Impacts of a Microwave-Assisted Extraction of Polyphenols and Comparison with an Ultrasound-Assisted Extraction Process. Foods.

[44] Mariatti F., Gunjević V., Boffa L., Cravotto G. (2021). Process Intensification Technologies for the Recovery of Valuable Compounds from Cocoa By-Products. Innov. Food Sci. Emerg. Technol..

[45] Sánchez M., Ferreira-Santos P., Gomes-Dias J.S., Botelho C., Laca A., Rocha C.M.R. (2023). Ohmic Heating-Based Extraction of Biocompounds from Cocoa Bean Shell. Food Biosci..

[46] Soria A.C., Villamiel M. (2010). Effect of Ultrasound on the Technological Properties and Bioactivity of Food: A Review. Trends Food Sci. Technol..

