# Supplementary material for: Improving the Extraction of Polyphenols from Cocoa Bean Shells by Ultrasound and Microwaves: A Comparative Study
Source: Antioxidants (Basel). 2024 Sep 10;13(9):1097. doi: 10.3390/antiox13091097 (PMC11428740; doi:10.3390/antiox13091097)
Supplement: Supplementary file 1 [file antioxidants-13-01097-s001.zip › Supplementary material.pdf]

Improving the extraction of polyphenols from cocoa bean shells by ultrasound and microwaves: a comparative study

Vincenzo Disca[1], Fabiano Travaglia[1], Chiara Carini[1], Jean Daniel Coïsson[1], Giancarlo Cravotto[2], Marco Arlorio[1*] and Monica Locatelli[1]

[1] Dipartimento di Scienze del Farmaco, Università del Piemonte Orientale "A. Avogadro", Via Bovio 6, 28100 Novara (Italy)

[2] Dipartimento di Scienza e Tecnologia del Farmaco, Università di Torino, Via P. Giuria 9, 10125 Torino (Italy)

Correspondence: fabiano.travaglia@uniupo.it

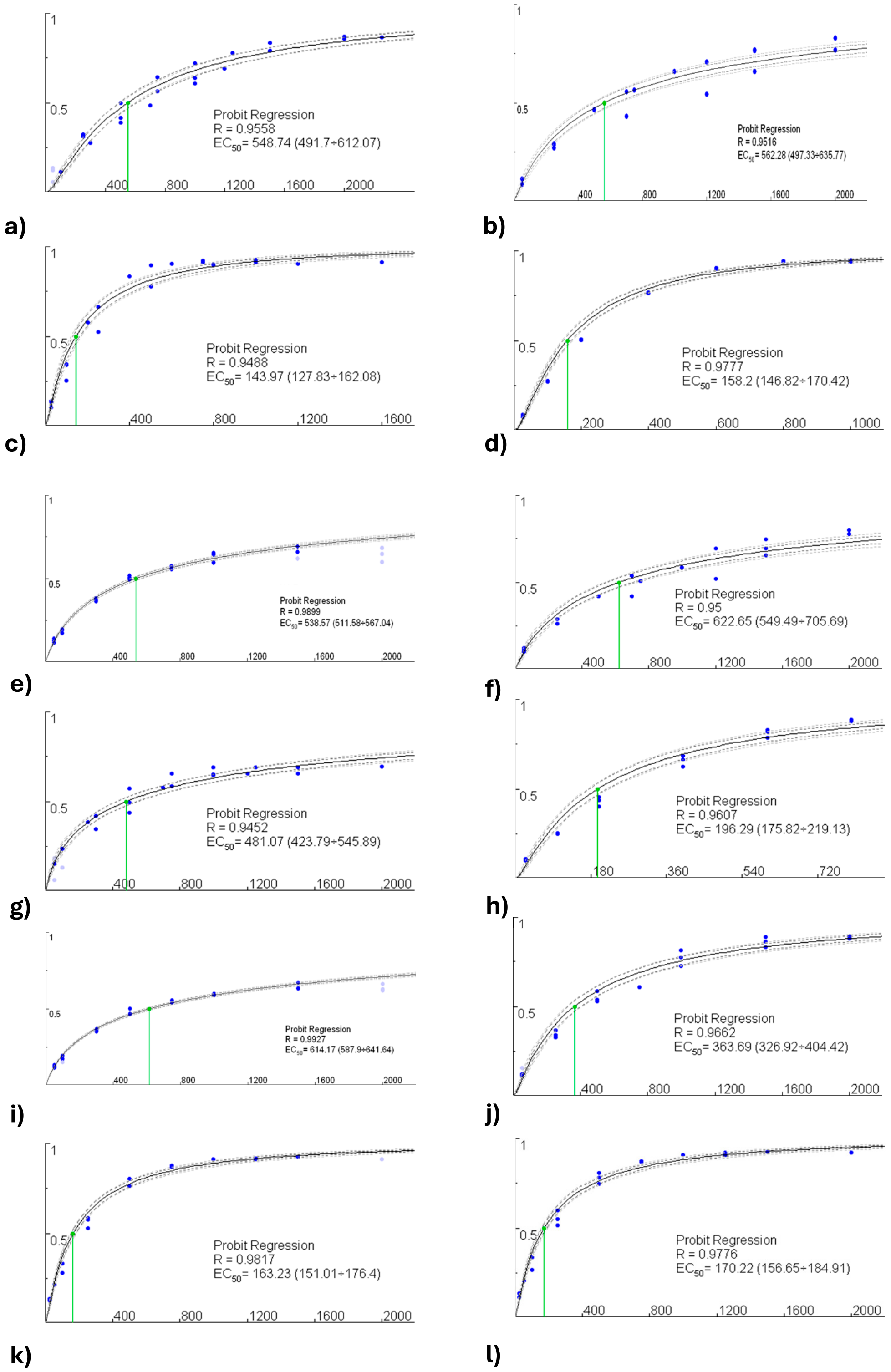

Figure S1. Probit regression curves for the different CBSs extracts: a) MS Raw; b) MS Defatted; c) SOX Raw; d) SOX Defatted; e) US Raw; f) US Defatted; g) MW Raw; h) MW Defatted; i) US/MW50 Raw; j) US/MW50 Defatted; k) US/MW90 Raw; l) US/MW 90 Defatted. Linearity and EC_{50} (µg/mL, CI 95%) are showed in each curve for each extract.

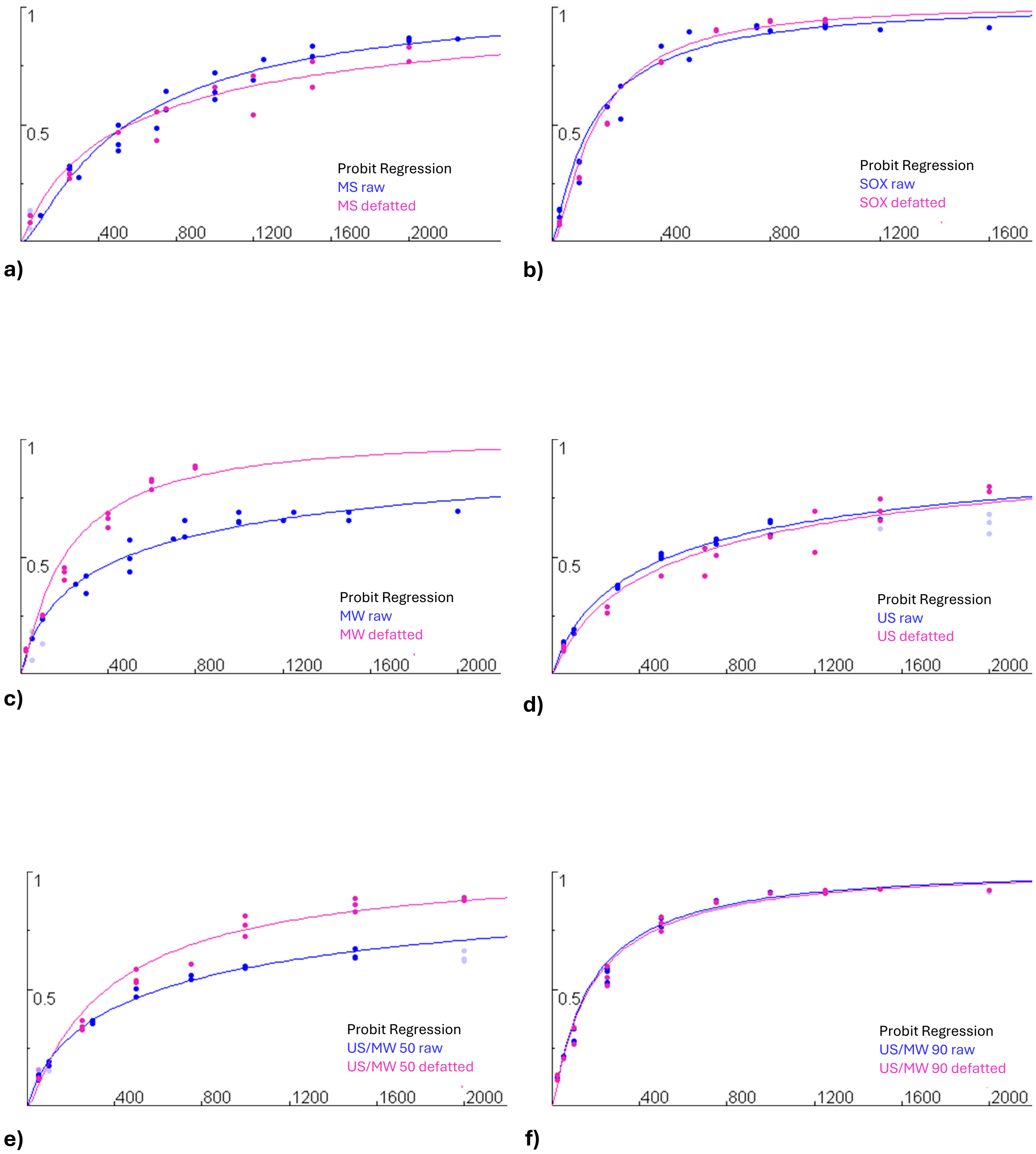

Figure S2. Comparison between the activity curves obtained from raw and those obtained for defatted CBS; curves characterized by p-values < 0.05 were considered significantly different.

a) Comparison of the antioxidant activity of MS Raw and MS Defatted extracts ($p < 0.001$);

b) Comparison of the antioxidant activity of SOX Raw and SOX Defatted extracts ($p < 0.05$);

c) Comparison of the antioxidant activity of MW Raw and MW Defatted extracts ($p < 0.\ 0001$);

d) Comparison of the antioxidant activity of US Raw and US Defatted extracts ($p > 0.05$);

e) Comparison of the antioxidant activity of US/MW50 Raw and US/MW50 Defatted extracts ($p < 0.0001$);

f) Antioxidant activity of US/MW90 Raw and US/MW90 Defatted extracts ($p > 0.05$).
